# Comparative intra-articular gene transfer of seven adeno-associated virus serotypes reveals that AAV2 mediates the most efficient transduction to mouse arthritic chondrocytes

**DOI:** 10.1371/journal.pone.0243359

**Published:** 2020-12-15

**Authors:** Quan Chen, Huan Luo, Chengcong Zhou, Huan Yu, Sai Yao, Fangda Fu, Rebecca Seeley, Xing Ji, Yanping Yang, Peifeng Chen, Hongting Jin, Peijian Tong, Di Chen, Chengliang Wu, Weibin Du, Hongfeng Ruan

**Affiliations:** 1 The First Affiliated Hospital of Zhejiang Chinese Medical University, Hangzhou, Zhejiang, China; 2 The First Clinical Medical College of Zhejiang Chinese Medical University, Hangzhou, Zhejiang, China; 3 Department of Pharmacy, The Second Affiliated Hospital, School of Medicine, Zhejiang University, Hangzhou, China; 4 Translational Research Program in Pediatric Orthopedics, The Children’s Hospital of Philadelphia, Philadelphia, PA, United States of America; 5 Longhua Hospital Affiliated to Shanghai University of Traditional Chinese Medicine, Shanghai, China; 6 Research Center for Human Tissues and Organs Degeneration, Shenzhen Institutes of Advanced Technology, Chinese Academy of Sciences, Shenzhen, China; 7 Research Institute of Orthopedics, the Affiliated JiangNan Hospital of Zhejiang Chinese Medical University, Hangzhou, China; Fudan University, CHINA

## Abstract

Osteoarthritis (OA) is the most common arthropathy, characterized by progressive degeneration of the articular cartilage. Currently, there are no disease-modifying approaches for OA treatment. Adeno-associated virus (AAV)-mediated gene therapy has recently become a potential treatment for OA due to its exceptional characteristics; however, the tropism and transduction efficiency of different AAV serotypes to articular joints and the safety profile of AAV applications are still unknown. The present study aims to screen an ideal AAV serotype to efficiently transfer genes to arthritic cartilage. AAV vectors of different serotypes expressing eGFP protein were injected into the knee joint cavities of mice, with all joint tissues collected 30 days after AAV injection. The transduction efficiency of AAVs was quantified by assessing the fluorescent intensities of eGFP in the cartilage of knee joints. Structural and morphological changes were analyzed by toluidine blue staining. Changes to ECM metabolism and pyroptosis of chondrocytes were determined by immunohistochemical staining. Fluorescence analysis of eGFP showed that eGFP was expressed in the cartilage of knee joints injected with each AAV vector. Quantification of eGFP intensity indicated that AAV2, 7 and 8 had the highest transduction efficiencies. Both toluidine blue staining and Mankin score showed that AAV6 aggravated cartilage degeneration. The analysis of key molecules in ECM metabolism suggested that AAV5 and 7 significantly reduced collagen type II, while AAV9 increased ADAMTS-4 but decreased MMP-19. In addition, transduction with AAV2, 5, 7 and 8 had no obvious effect on pyroptosis of chondrocytes. Comprehensive score analysis also showed that AAV2 had the highest score in intra-articular gene transfer. Collectively, our findings point to AAV2 as the best AAV serotype candidate for gene transfer on arthritic cartilage, resulting in minimal impact to ECM metabolism and pyroptosis of chondrocytes.

## Introduction

Osteoarthritis (OA) is one of the most common forms of arthropathy, affecting more than 10% of adults with higher rate in women than in men, and provoking considerable pain and disability [1, 2]. Currently available studies attribute the pathogenesis of OA partially to dysregulation of extracellular matrix (ECM) metabolism and the reduction of chondrocytes [3]. Despite considerable improvement in the pharmacological treatment of OA [4], the above challenges indicate that innovative strategies remain warranted. Growing numbers of studies have focused on gene therapy of OA by correcting or supplementing defective genes. There are mainly two classes of expression vectors in gene transfer: viral and non-viral. Viral vectors include: adenovirus, recombinant adeno-associated virus (AAV), retroviruses and baculoviruses. Recently, significant attention has been focused on delivering all types of genes using AAV vectors in animal models, while some serotype of AAV vectors have been used in clinical trials to evaluate use as human gene therapy, including joint diseases [5–7].

AAV represents the most attractive platform for viral-mediated gene therapy and is also a valuable research tool for studying gene function or establishing disease models, as it shows minimal pathogenicity in humans and can provide long-term gene expression [8]. In 2012, Glybera, an AAV vector designed to express lipoprotein lipase, became the first gene therapy product approved for patients with lipoprotein lipase deficiency [9], a milestone achievement in the development of AAV therapy. At present, at least 10 AAV serotypes are commercially available and used in preclinical research (AAV1-9 and AAV-DJ). The tissue tropism and abundance of gene expression depends on the specific AAV serotype and the tissue to which it is applied. Some laboratories have compared the arthritic gene transfer efficiency of different AAV serotypes (AAV1-5 or AAV1-2, 5, 8, 9), and found that intra-articular injection of AAV5 serotype resulted in the highest synovial transduction [10, 11]. However, the differences in gene transduction efficiency between these serotypes and newly established AAV serotypes (AAV6, 7, and AAV-DJ) into articular cartilage remains unquantified. In addition, the effect of application of these AAV serotypes on ECM metabolism and pyroptosis of chondrocytes remains unknown. Therefore, screening an optimal AAV serotype for transgene expression into arthritic cartilage is important for arthritic joints in preclinical research.

Articular cartilage is composed of dense ECM with dispersed highly specialized cells (chondrocytes). The metabolic imbalance of ECM is a key factor in the development and progression of OA. Chondrocytes can not adhere to defective ECM, leading to the degeneration of articular cartilage [12, 13]. Type II collagen (Col2) and Aggrecan are the primary structural components of cartilage ECM, thus their degradation is relevant to the development of OA. MMPs are a family of zinc-dependent enzymes; MMP-3 and MMP-13 are known as the principal collagenases responsible for the degradation of Col2 [14]. ADAMTS-4 and ADAMTS-7 are members of the ADAMTS family, which function as Aggrecanase and participate in the degradation of Aggrecan. ADAMTS-7 can also form a positive feedback loop with TNF-α and further upregulates the expression of MMP-3, MMP-13 and ADAMTS-4, resulting in accelerated OA progression [15, 16]. Additionally, MMP-19 may also participate in the degeneration of Aggrecan and cartilage oligomeric matrix protein [17].

Pyroptosis, also known as inflammatory necrosis, is a new form of programmed inflammatory cell death, requiring the formation of a large supramolecular complex termed the inflammasome and auto-activation of the enzyme Caspase-1 [18]. During the process of pyroptosis, activated nod-like receptor protein 3 (NLRP3) combines with apoptosis-associated speck-like protein (ASC) to form inflammasome bodies, which further induce the recruitment of pro-Caspase-1. Subsequently, Caspase-1 is activated following cleavage of pro-Caspase-1 and released from the inflammasome, further inducing pyroptosis by Gasdermin D and promoting the maturation of IL-1β [18]. Recently, we and other researchers have found that induction of pyroptosis in fibroblast-like synoviocytes and articular chondrocytes is related to the pathogenesis of OA [19, 20]. Gene intervention on the NLRP3-inflammasome and pyroptosis may therefore be a promising therapeutic target for OA.

The goal of this study is to screen for a successful and reliable AAV vector serotype for gene modification in mouse articular chondrocytes. Seven commonly used serotypes of AAV encoding eGFP (with same titer 5 × 10^12^ vg/mL) were injected into knee joint cavities of C57BL/6J mice. We compared the tropism and transduction efficiency of AAV vectors of different serotypes by quantifying the expression of eGFP in articular cartilage. Changes to the ECM and related catabolic enzymes were analyzed by IHC to evaluate the effects of AAVs on ECM metabolism and pyroptosis of chondrocytes. Finally, we found AAV2 to be the ideal AAV serotype of those screened for gene transfer into knee cartilage, which has minimal impact on ECM metabolism and pyroptosis of chondrocytes.

## Materials and methods

### Chemicals and reagents

AAV5 and AAV-DJ were obtained from Hanbio Biotechnology (Shanghai, China), and AAV2/6/7/8/9 were obtained from Vigene Biosciences (Shandong, China). All serotypes of AAV encoding eGFP protein under the cytomegalovirus promoter and the viral genome titer were optimized as 5 × 10^12^ vg/mL. Primary antibodies against Col2 (RLT1022), MMP-3 (RLT4465), MMP-19 (RLT2797), NLRP3 (RLT5382), Caspase-1 (RLT0652) were obtained from Ruiying Biological (Jiangsu, China). Primary antibodies against ADAMTS-4 (PA001311LA01HU), ADAMTS-7 (PA891951LA01HU) were obtained from CUSABIO BIOTECH Co. (Wuhan, China). Primary antibodies against MMP-13 (ab39012), Aggrecan (ab36861) were obtained from Abcam (Cambridge, UK). Unless otherwise mentioned, all chemicals were obtained from Sigma-Aldrich (St. Louis, MO).

### Animals and treatments

Eight-week-old C57BL/6 male mice (20 ~ 22 g) were obtained from Zhejiang Chinese Medical University laboratory animal research center (Grade SPF, SCXK (Shanghai): 2017–0005). All mice were housed under controlled pathogen-free conditions with a 12 h light/dark cycle, with free access to rodent chow and autoclaved water. All animal experimental procedures were approved by the Ethics Committee of Zhejiang Chinese Medical University (Protocol Number: ZSLL-2018-011). All operations were performed under isoflurane anesthesia, with all efforts made to minimize suffering.

Mice (n = 50) were randomly divided into 9 groups, including a Control Group (n = 10), seven AAV groups (n = 5 in each group), and an OA model group (n = 5). Anesthetized mice (1.5% isoflurane) in each AAV group were subjected to a single injection of 6 μl of the group’s specific AAV serotype into the right knee joints, while mice in the Control group and OA model group were injected with an equal volume of normal saline. All injections were performed using a 10 μL-microinjector (1701 RN) and a 32-gauge needle (7803–04), obtained from Hamilton Company (Reno, NV). Mice in the OA model group received medial meniscus destabilization surgery (DMM surgery) to prepare the OA model, as previously described [20].

**Fig 1:**
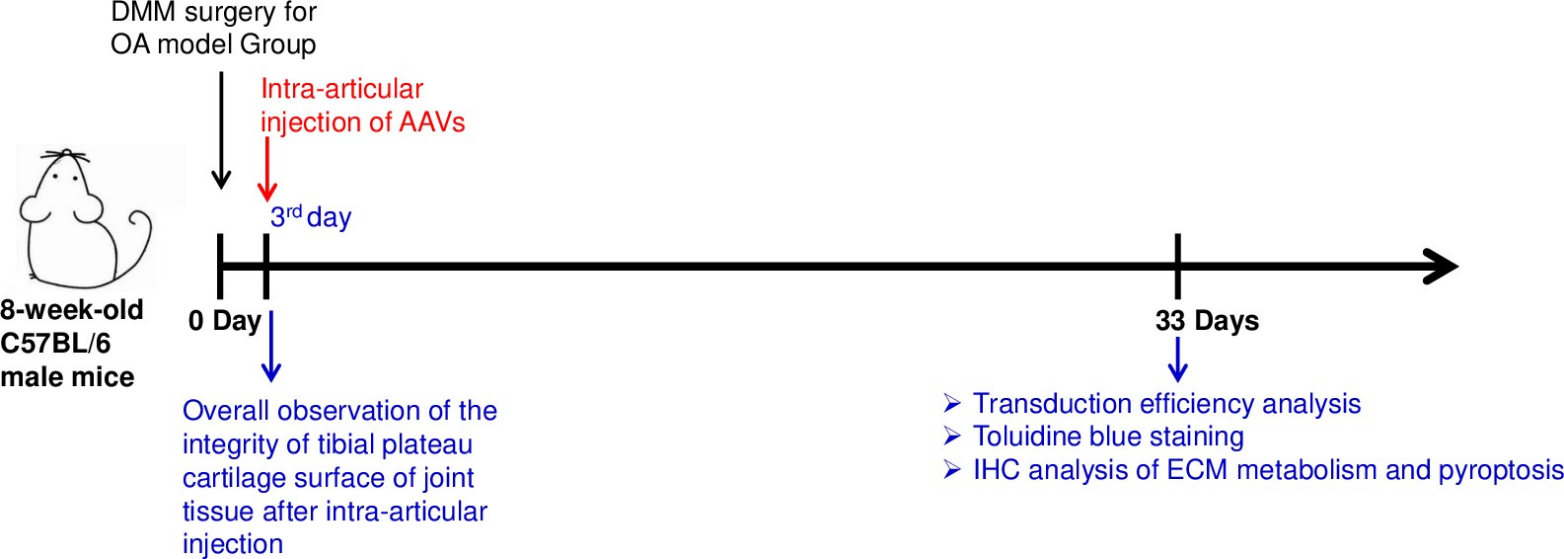
Animal treatment and study design of the project.

Five mice in the Control group were directly sacrificed after injection, and the joint tissues were observed under a stereomicroscope (Zeiss stemi 2000 c) to determine whether intra-articular injection resulted in joint tissue damage. The remaining mice were sacrificed 30 days after AAV injection, and the knee joint tissues were collected for further experiments (Fig 1). Knee joint tissues were fixed in 4% polyformaldehyde for 24 h, decalcified in 10% EDTA for 14 days, and dehydrated in 10% ~ 30% gradient sucrose solution until tissue precipitated to the bottom of the test tube. After being embedded in OCT (Sakura Finetechnical, Tokyo, Japan), 10-μm-thick sections were cut along the sagittal plane.

### Transduction efficiency analysis

Sections from all AAV groups and the Control group were observed under the Zeiss fluorescence microscope (Carl Zeiss, Göttingen, Germany), and the intensities of eGFP fluorescence in the cartilage area of tibial plateaus of arthritic joints were quantified by a blind method using Image J software (Image J, NIH, Bethesda, MD), with the mean intensity in the Control group set to 1.

### Histological and immunohistochemical (IHC) analysis

Gross structural observation and Toluidine Blue staining were performed to evaluate the damage of arthritic cartilage as previously described [21]. The severity of articular cartilage degeneration was graded on a scale of 0–13, according to the modified Mankin scoring system [22]. Histopathological evaluation was conducted by two independent blind observers, and the average value of the data obtained by the two observers was used to calculate the Mankin score.

IHC analysis was performed by using SP Link Detection Kits (ZSGB-BIO, Beijing, China). Briefly, after deparaffinizing in xylene and rehydration in a series of ethanol baths, sections were treated with sodium citrate (0.1 mol/L, pH = 6.0) for antigen retrieval and 3% H_2_O_2_ to reduce endogenous peroxidase activity. Subsequently, the sections were blocked with normal goat serum and further incubated with primary antibodies, including Col2 (1/400), MMP-3 (1/800), MMP-13 (1/800), Aggrecan (1/800), ADAMTS-4 (1/150), ADAMTS-7 (1/200), MMP-19 (1/300), Caspase-1 (1/100), NLRP3 (1/400) overnight at 4 C. The following day, they were incubated with HRP-conjugated secondary antibody and the reaction was observed with diaminobenzidine followed by counterstaining with hematoxylin. The images were captured using the Zeiss microscope, and the optical densities of target protein-positive cells were quantified using Image J software. The average optical density value of each sample was obtained by dividing the total statistical area. The relative optical density value of each group was then normalized to the average intensity of the Control group.

### Statistical analysis

All data were expressed as mean ± SD. Statistical differences between the Control group and other groups were tested using a one-way analysis of variance (ANOVA) followed by Dunnett’s test (GraphPad Software Inc., La Jolla, CA). Statistical significance was assessed at *P* < 0.05 and *P* < 0.01.

## Results

### AAV2 serotype has the highest transfection efficiency to articular cartilage

**Fig 2:**
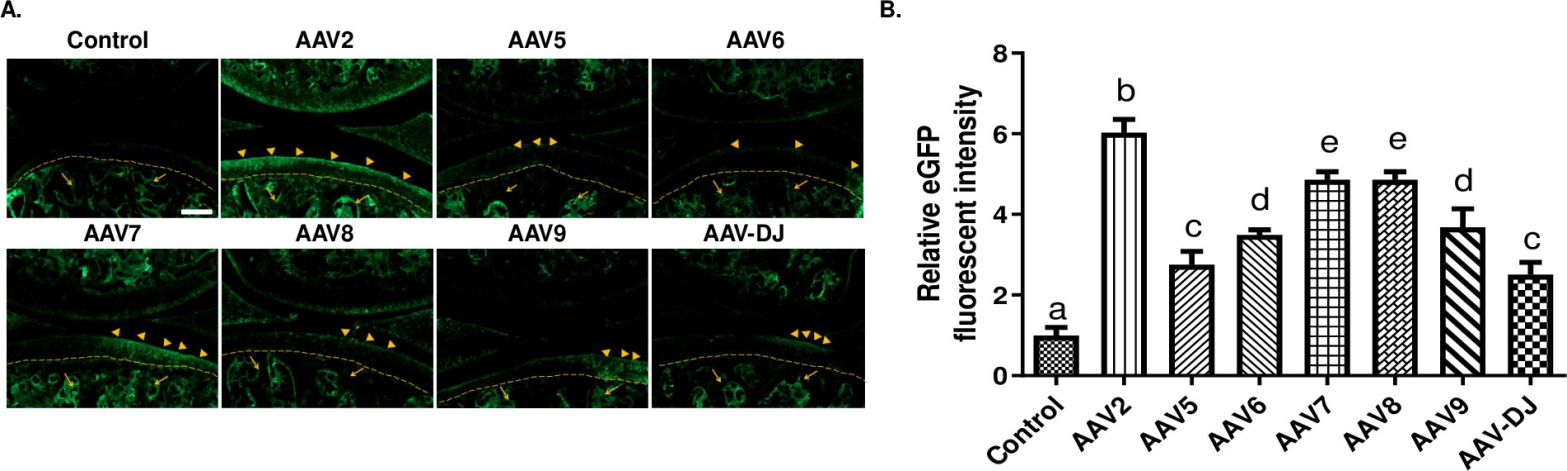
eGFP was expressed in mouse cartilage tissue 30 days after intra-articular injection of different AAV serotypes. (**A**) The expression of eGFP in mouse cartilage tissue was observed with an inverted fluorescence microscope. Yellow arrowhead, eGFP expression in articular cartilage; Yellow arrow, non-specific fluorescence in the subchondral bone. n = 5, Scale bar = 200 μm. (**B**) The density of eGFP in arthritic cartilage in **A**. Data are expressed as mean ± SD. Bars in **B** sharing a *common letter* are not significantly different (*P* ≥ 0.05) between groups.

To compare the tropism and transduction efficiency of these seven commonly used AAV serotypes (including AAV 2, 5, 6, 7, 8, 9 and AAV-DJ) to articular cartilage *in vivo*, we injected an equal volume of different AAV serotypes (3 × 10^10^ vg particles in 6 μL) into the knee joint cavities of mice. The transfection efficiency was evaluated 30 days after AAV injection using eGFP protein as a reporter of transgene expression. We found that eGFP was specifically expressed in the articular cartilage, synovium, and menisci of all AAV-injected mice, but not in those of Control mice (Fig 2A). Quantification of eGFP fluorescent intensity showed that the average expression level of eGFP in the cartilage area of each AAV mouse was strikingly higher than that of the Control mice (the fold difference ranged from 2.5- to 6-folds. Compared with the other tested AAVs, the arthritic cartilage of AAV2 mice had the highest eGFP intensity (transduction efficiency in decreasing order, AAV2 > AAV7 ≈ AAV8 > AAV 9 ≥ AAV6 > AAV5 > AAV-DJ) (Fig 2B), indicating that the AAV2 serotype has a stronger affinity for articular cartilage than other AAV serotypes.

### AAV6 promotes proteoglycan loss

**Fig 3:**
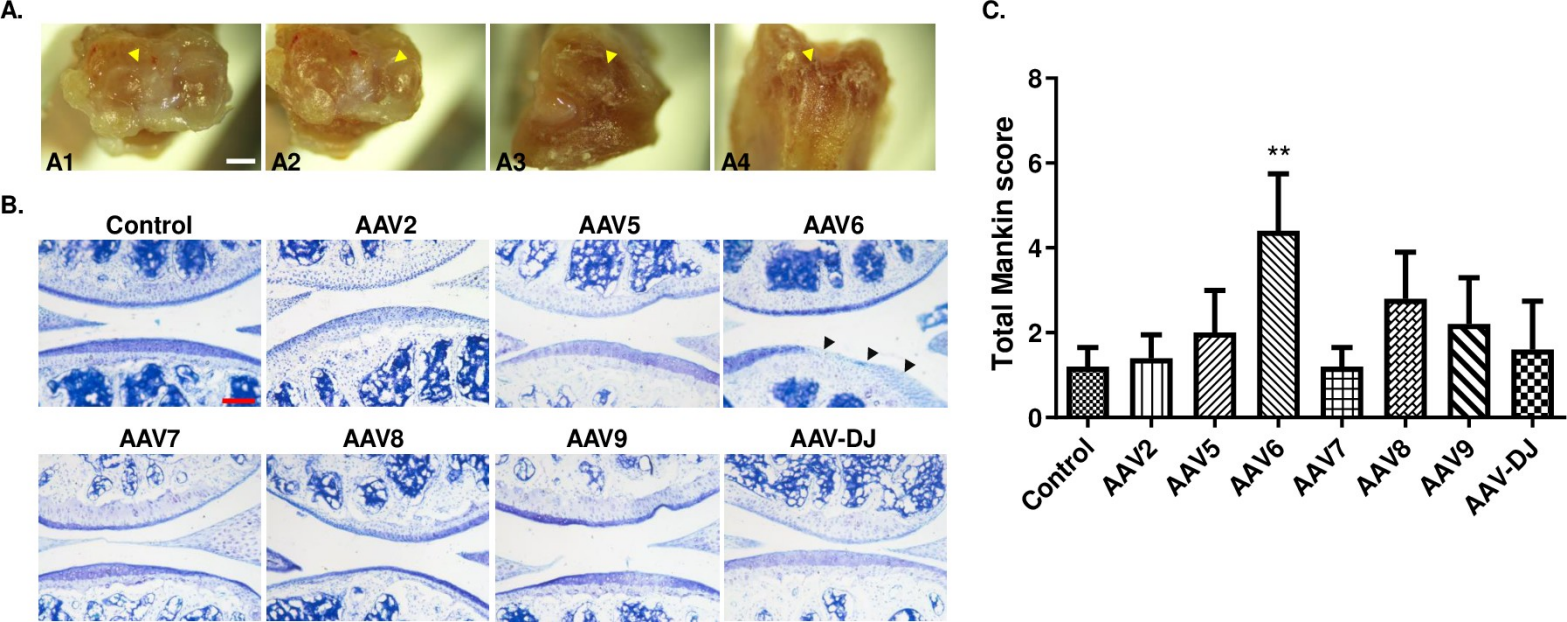
Gross appearance and histological changes of knee joints. (**A**) After intra-articular injection, the knee joints were directly dissected and then tibial plateau (**A1**), meniscus (**A2**), femoral condyles (**A3**), femoral trochlea (**A4**) were observed under a stereomicroscope. n = 5, Scale bar = 0.8 mm. (**B**) The knee joints were dissected 30 days after AAV injection, and the cartilage area of the medial tibial plateaus was stained with toluidine blue. n = 5, Scale bar = 200 μm. (**C**) Degeneration of articular cartilage was evaluated using the classical Mankin scoring system. Black arrowhead, positive staining of MMPs in articular cartilage. Data are expressed as mean ± SD. *P < 0.05, ** *P* < 0.01.

To assess whether intra-articular injection causes adverse effects on articular cartilage, five knee joints of mice in the Control group were directly dissected after intra-articular injection, and surface integrity of articular cartilage was analyzed under a stereomicroscope. We found that the surface of tibial plateaus was smooth without visible defect, such as discoloration of tissues or cartilage erosion; the menisci were complete with moderate thickness; femoral condyles were complete and smooth, without osteophytes; and femoral trochleae were moderate in depth without any injuries (Figs 3A1 and 4). These results indicate that there is no obvious damage to the knee joint tissues with use of intra-articular AAV injection.

**Fig 4:**
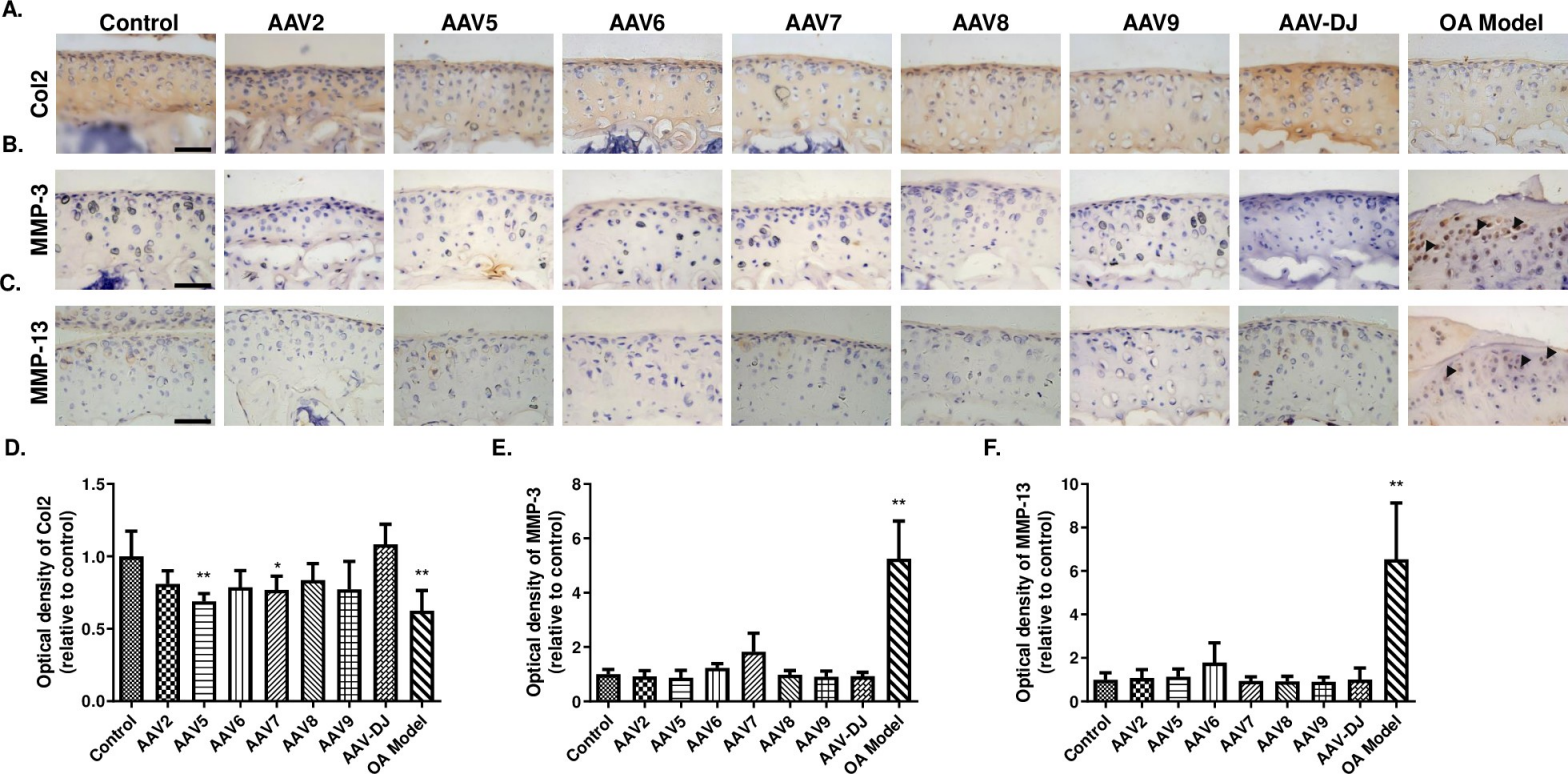
Immunohistochemical analysis of Col2, MMP-3, MMP-13 expression in the cartilage of knee joints. (**A**-**C**) Immunohistochemical staining results of Col2 (**A**), MMP-3 (**B**) and MMP-13 (**C**) in articular cartilage. (**D**-**F**) The relative optical density of positive staining in **A**-**C**. Col2, Type II collagen; MMP-3, Matrix metalloproteinase-3; MMP-13, Matrix metalloproteinase-3. Data are expressed as mean ± SD. *P < 0.05, **P < 0.01. n = 5. Scale bar = 50 μm.

To further confirm the effects of intra-articular injection on arthritic joints, sections of knee joints were stained with toluidine blue to evaluate changes to proteoglycan content in the cartilage. The resulting morphological staining showed that AAV6 promoted regional loss of proteoglycan in cartilage (Fig 3B). The Mankin score system was applied to evaluate the severity of this loss. We found that the AAV6 mice had a significantly higher score than the Control mice (4.4 ± 1.3 *vs* 1.2 ± 0.4), and a non-significant or much milder increase of Mankin score was also observed in arthritic cartilage of mice receiving AAV5, 8, or 9 (Fig 3C).

### AAV5 and AAV7 decrease the expression of Col2

To test whether AAV injection affects the metabolism of Col2, IHC analysis for Col2 and its collagenases (MMP-3 and MMP-13) was performed. The OA model group was used as a positive Control group, displaying changes to ECM metabolism as well as pyroptosis of chondrocytes [20]. We found that the expression of Col2 in the OA model group was remarkably decreased. Similarly, AAV5 and 7 treatment significantly decreased the expression of Col2 in articular cartilage (Fig 4A and 4D). Interestingly, despite the significantly increased expression of MMP-3 and MMP-13 in the OA model group, there was no significant difference in MMP-3 and MMP-13 expression between the Control group and other AAV groups, except for a non-significant increase of MMP-3 in AAV7 mice and a non-significant increase of MMP-13 in AAV6 mice (Fig 4B, 4C, 4E and 4F).

### AAV5 and AAV9 affect the degradation of Aggrecan

**Fig 5:**
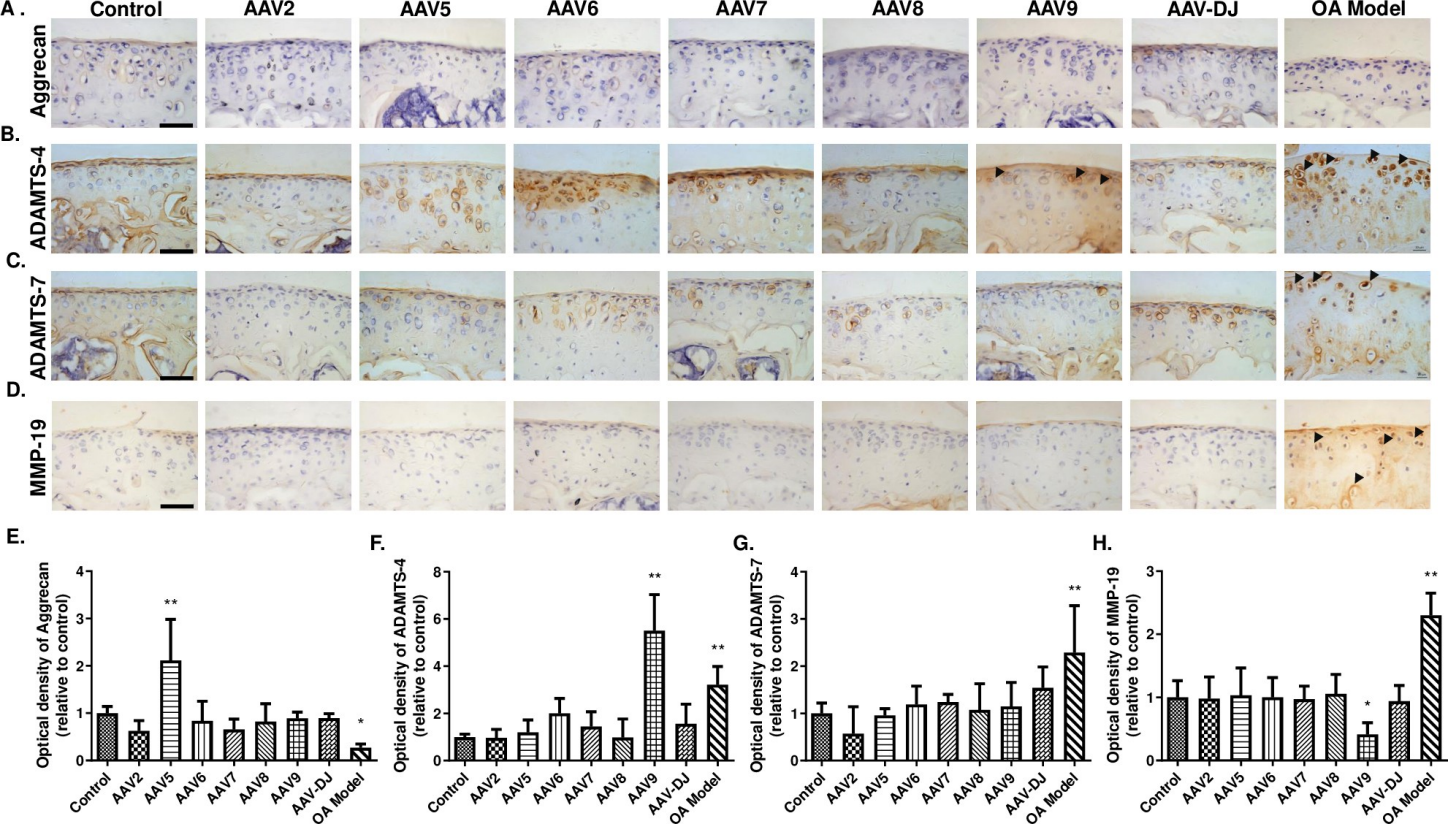
Immunohistochemical analysis of Aggrecan, ADAMTS-4, ADAMTS-7 and MMP-19 expressions in the articular cartilage. (**A**-**D**) Immunohistochemical staining of Aggrecan (**A**), ADAMTS-4 (**B**), ADAMTS-7 (**C**), MMP-19 (**D**) expressions in articular cartilage. (**E**-**H**) The relative optical density of positive staining in **A**-**D**. ADAMTS, A disintegrin and metalloproteinase with thrombospondin motifs; MMP-19, Matrix metalloproteinase-19. Black arrowhead, positive staining of Aggrecanases in articular cartilage. Data are expressed as mean ± SD. *P < 0.05, **P < 0.01. n = 5. Scale bar = 50 μm.

Next, to evaluate whether AAV injection affects the metabolism of Aggrecan (another important matrix in cartilage), IHC analysis of Aggrecan, ADAMTS-4, ADAMTS-7, and MMP-19 was carried out in all groups. Compared to the Control group, we found Aggrecan was remarkably decreased in the OA model group, with ADAMTS-4, ADAMTS-7, and MMP-19 levels significantly increased (Fig 5A–5H). Unexpectedly, AAV5 injection significantly upregulated the level of Aggrecan (Fig 5A and 5E), while AAV9 treatment resulted in the elevation of ADAMTS-4 and the reduction of MMP-19 (Fig 5B, 5D, 5F and 5H).

### AAVs mediated gene transfer has no obvious effect on pyroptosis of chondrocytes

**Fig 6:**
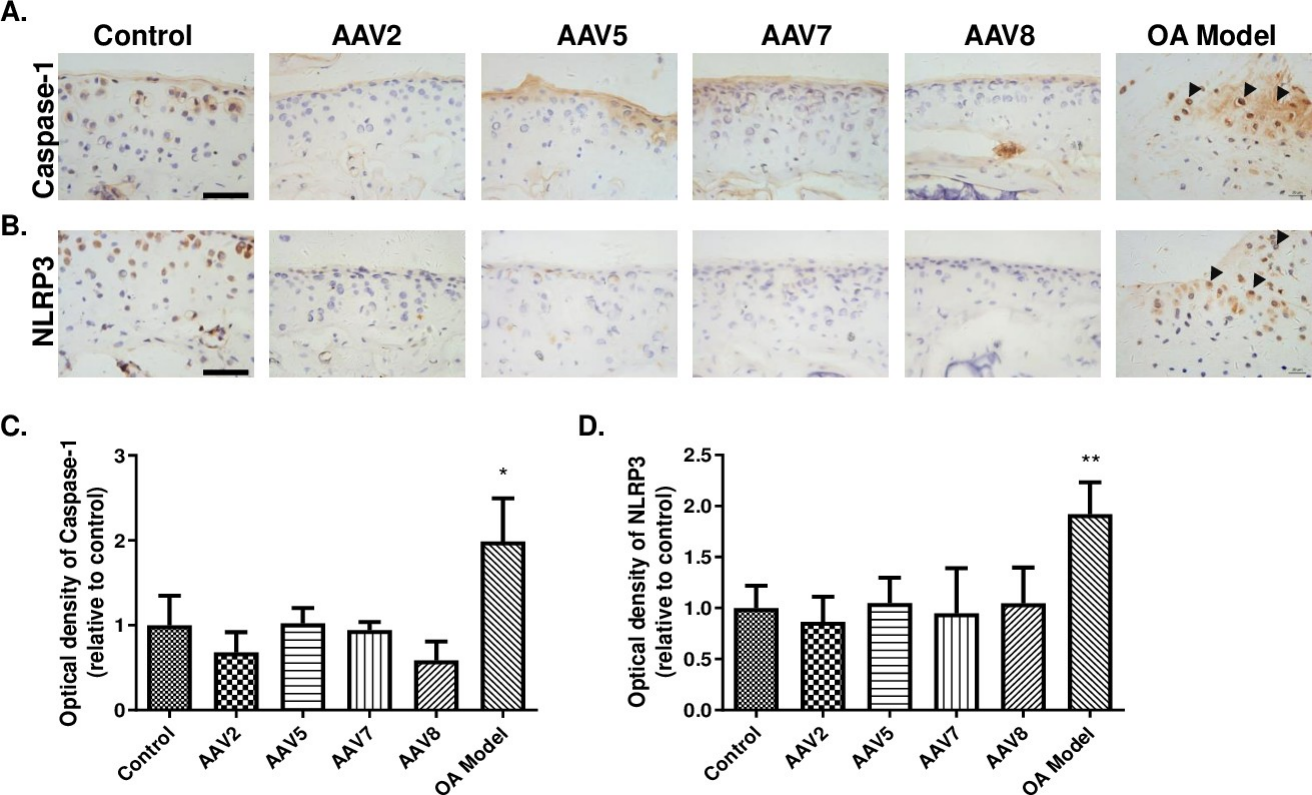
Immunohistochemical analysis of pyroptosis of the articular cartilage. (**A**-**B**) Immunohistochemical staining of Cryopyrin (**A**) and Caspase-1 (**B**). (**C**-**D**) The relative optical density of positive staining in **A**-**B**. Cryopyrin (NLRP3), Nucleotide-binding oligomerization domain-like receptor family pyrin domain-containing protein 3; Caspase-1, Cysteinyl aspartate specific proteinase-1. Data are expressed as mean ± SD. *P < 0.05, **P < 0.01. n = 5. Scale bar = 50 μm.

Our previous study suggested DMM surgery-induced OA Modeling promotes pyroptosis of chondrocytes concomitant with elevated expression of Caspase-1 and NLRP3 proteins in chondrocytes [20]. Considering the high transduction efficiency of AAV2, 7, and 8 in cartilage; we further analyzed the influence of these 3 AAV serotypes on pyroptosis of chondrocytes. Consistent with our previous study [20], immunostaining results showed remarkable increases of Caspase-1 and NLRP3 proteins in the OA model group (Fig 6A–6D), whereas no significant differences in Caspase-1 and NLRP3 expression were found between AAV mice and Control mice (Fig 6A–6D). As numerous preclinical studies have used AAV5 serotype to deliver genes for arthritis treatment [3, 11, 23, 24], we also examined the effect of AAV5 injection on pyroptosis of chondrocytes. Consistent with the result of the above 3 AAV serotypes, AAV5 showed no effect on the pyroptosis of chondrocytes (Fig 6A–6D).

### AAV2 may be the best candidate serotype for gene transfer to arthritic joints

To compare the transduction efficiency and safety of different AAV serotypes more intuitively and comprehensively, we designed a scoring standard to compare the comprehensive performance of all AAV serotypes in gene transfer to articular chondrocytes (Table 1). The total score of each AAV serotype with or without their contributions to pyroptosis of chondrocytes (named as Score-1 or Score-2) was calculated. Comparison results of Score-1 showed that the subscore that made the biggest difference was Mankin scores and AAV2 received the highest score. Similar results were also obtained from the comparison results of Score-2 (Table 2), indicating that the AAV2 serotype might be our best serotype candidate for treating arthritic joint diseases.

**Table 1:** Scoring criteria of all AAV serotypes in gene transfer to articular chondrocyte.

Item	Scoring criteria
**Fluorescence intensity of eGFP (0–6)**	Relative fluorescence intensity ≤ 1, scores 0 point; > 1 and ≤ 2, scores 1 point; > 2 and ≤ 3, scores 2 points; > 3 and ≤ 4, scores 3 points; > 4 and ≤ 5, scores 4 points; > 5 and ≤ 6, scores 5 points; > 6, scores 6 points.
**Mankin scores (0–6)**	Total Mankin scores> 4, scores 0 point; > 3.5 and ≤ 4, scores 1 point; > 3 and ≤ 3.5, scores 2 points; > 2.5 and ≤ 3, scores 3 points; > 2 and ≤ 2.5, scores 4 points; > 1.5 and ≤ 2, scores 5 points; ≤ 1.5, scores 6 points
**Col2 (0–3)**	Col2 is the main structural matrix in articular cartilage. Tissues with higher levels of Col2 have higher scores.
	Relative optical density: ≤ 0.7, scores 0 point; > 0.7 and ≤ 1, scores 1 point; > 1 and ≤ 1.3, scores 2 points; > 1.3, scores 3 points
**MMP-3 (0–3)**	MMP-3 is an important collagenase involved in the degradation of articular cartilage matrix. Tissues with higher levels of MMP-3 have lower scores.
	Relative optical density: > 2, scores 0 point; > 1.5 and ≤ 2, scores 1 point; > 1 and ≤ 1.5, scores 2 points; ≤ 1, scores 3 points
**MMP-13 (0–3)**	MMP-13 is the most studied matrix metalloproteinase that cleaves Col2 in the articular cartilage. Tissues with higher levels of MMP-13 have lower scores.
	Relative optical density: > 2, scores 0 point; > 1.5 and ≤ 2, scores 1 point; > 1 and ≤ 1.5, scores 2 points; ≤ 1, scores 3 points
**Aggrecan (0–3)**	Aggrecan is a major component of ECM in articular cartilage. Tissues with higher levels of Aggrecan have higher scores.
	Relative optical density: ≤ 0.5, scores 0 point; > 0.5 and ≤ 1, scores 1 point; > 1 and ≤ 1.5, scores 2 points; > 1.5, scores 3 points
**ADAMTS-4 (0–3)**	ADAMTS-4 is an aggrecanase capable of inducing aggrecan degradation, therefore tissues with high ADAMTS-4 receive lower scores.
	Relative optical density: > 2, scores 0 point; > 1.5 and ≤ 2, scores 1 point; > 1 and ≤ 1.5, scores 2 points; ≤ 1, scores 3 points
**ADAMTS-7 (0–3)**	ADAMTS-7 is another member of ADAMTS associated with the degradation of Aggrecan. Tissues with higher levels of ADAMTS-7 have lower scores.
	Relative optical density: > 2, scores 0 point; > 1.5 and ≤ 2, scores 1 point; > 1 and ≤ 1.5, scores 2 points; ≤ 1, scores 3 points
**MMP-19 (0–3)**	MMP-19 is an enzyme involved in the degradation of various components of ECM in articular cartilage. Tissues with higher levels of MMP-19 have lower scores.
	Relative optical density: > 1.5, scores 0 point; > 1 and ≤ 1.5, scores 1 point; > 0.5 and ≤ 1, scores 2 points; ≤ 0.5, scores 3 points
**NLRP3 (0–3)**	NLRP3 is a critical regulator of pyroptosis which plays a key role in mediating the pathogenesis of OA. Tissues with higher levels of NLRP3 have lower scores.
	Relative optical density: > 2, scores 0 point; > 1.5 and ≤ 2, scores 1 point; > 1 and ≤ 1.5, scores 2 points; ≤ 1, scores 3 points
**Caspase-1 (0–3)**	Caspase-1 is a cysteine protease that induces pyroptosis after its activation by various inflammasomes. Tissues with higher levels of Caspase-1 have lower scores.
	Relative optical density: > 2, scores 0 point; > 1.5 and ≤ 2, scores 1 point; > 1 and ≤ 1.5, scores 2 points; ≤ 1, scores 3 points

To make the experimental results more intuitive and comprehensive, we designed a scoring standard to compare the comprehensive performance of all AAV serotypes in gene transfer to articular chondrocytes. The higher the comprehensive score, the more suitable the AAV serotype was for the transfection of articular cartilage. The transformation efficiency of eGFP and Mankin scores were taken as basic indicators, accounting for 0–6, while the single score of immunohistochemistry is 0–3.

**Table 2:** Comprehensive scores of different AAV serotypes for gene transfer to articular chondrocytes.

Serotype Item	AAV2	AAV5	AAV6	AAV7	AAV8	AAV9	AAV-DJ
Intensity of eGFP	6	2	3	4	4	3	2
Mankin scores	6	5	0	6	3	4	5
Col2	1	0	1	0	1	1	2
MMP-3	3	3	2	1	3	3	3
MMP-13	2	2	1	3	3	3	2
Aggrecan	1	3	1	1	1	1	1
ADAMTS-4	3	2	1	2	3	0	1
ADAMTS-7	3	3	2	2	3	2	1
MMP-19	2	1	2	2	2	3	2
**Total score 1**	**27**	**21**	**13**	**21**	**23**	**20**	19
NLRP3	3	2	/	3	2	/	/
Caspase-1	3	2	/	3	3	/	/
**Total score 2**	**33**	**25**	**/**	**27**	**28**	**/**	**/**

eGFP, green fluorescent protein; Col2, Type II collagen; MMP, Matrix metalloproteinase; ADAMTS, a disintegrin and metalloproteinase with thrombospondin motifs; NLRP3, activated nod-like receptor protein 3; Caspase-1, Cysteinyl aspartate specific proteinase-1. /, no influence.

## Discussion

Many viral vectors, including AAV, retrovirus, lentivirus (a subtype of retrovirus) and adenovirus, have been investigated as methods to induce overexpress of important proteins for the management of OA [25]. AAV is rapidly becoming the preferred vector for gene therapy *in vivo*, because it can transduce dividing and nondividing cells, enhancing the effectiveness of treatment, and allowing for long-term expression without inducing significant immune response or non-associated disease [26, 27]. The discovery of new AAV serotypes enables us to identify AAV serotypes that are more suitable for use in genetic modification of arthritic chondrocytes, which is crucial for screening potential effective approaches for treating arthritic disease. Until now, to our knowledge, few studies have focused on the comparison of transduction efficiency and safety of AAV-mediated gene transfer into mouse arthritic chondrocytes. In this study, we compared the transduction efficiency of seven widely used AAV serotypes coding eGFP protein to the mouse articular cartilage, as well as their effects on the metabolism of ECM and the pyroptosis of chondrocytes. Our findings showed that, compared with other AAV serotypes, intra-articular injection of AAV2 resulted in the highest transduction efficiency to arthritic cartilage and better comprehensive score, indentifying that AAV2 as the most ideal AAV serotype for gene transfer to arthritic joints, concomitant with minimal impacts on ECM metabolism and pyroptosis of arthritic chondrocytes. In addition, AAV5, 6, 7 and 9 had more adverse effects on the integrity of articular cartilage or ECM metabolism. Together, our data provides molecular evidence which proves that AAV2 vector-mediated gene modification of articular cartilage is a potential method for treatment of arthritic diseases.

An earlier comparative study of arthritic gene delivery using AAV1-5 encoding β-galactosidase showed that direct intra-articular injection of the AAV5 resulted in high synovial transduction, as indicated by the β-galactosidase staining and gDNA analysis of the AAV injected joints. These results were coupled with much lower expression using AAV2, whereas AAV1, 3 and 4 resulted in undetectable expression [10]. Therefore, in the present study, we only compared the transduction efficiency and safety of AAV2 and AAV5 with other newly established and commercially available serotypes, AAV6-9 and AAV-DJ, but not with AAV1, 3 and 4. In agreement with the previous study in rats and equines [10, 28], we found that serotypes 2 and 5 can successfully transduce eGFP expression in menisci and cartilage of knee joints. However, quantification of eGFP fluorescent intensity suggested AAV2 had a higher transduction efficiency than other AAVs (transduction efficiency in decreasing order, AAV2 > AAV7 ≈ AAV8 > AAV 9 ≥ AAV6 > AAV5 > AAV-DJ). This discrepancy may be ascribed to the higher sensitivity of fluorescence detection versus β-galactosidase staining, as well as differences in other experimental conditions, such as different species, methodologies, titers and sources of AAV vectors. Furthermore, AAV2.5, a mutant vector of AAV2, showed higher transfection efficiency than AAV5 on human and equine articular fibroblasts and synoviocytes [11, 29, 30], indicating AAV2 serotype mediated the most efficient transduction to mouse arthritic chondrocytes.

Additionally, serotype-specific affinities of internalization receptors on the cell surface may partially account for the differences in transduction efficiency to the cartilage of mice [30, 31]. It has been found that AAV serotypes bind with different receptors on the host cell surface, and some serotypes may bind to a secondary receptor, so as to promote virus endocytosis into endosomes after binding to the primary receptor [32]. The primary receptors identified so far include heparan sulfate proteoglycan (for AAV2, 3, 6 and AAV-DJ) [33], specific N- or O-linked sialic acid moieties (for AAV1, 4, 5, and 6), and N-terminal galactose (for AAV9) [32, 34, 35]. Secondary receptors include 37/67-kDa laminin receptor (for AAV2, 3, 8 and 9), fibroblast growth factor receptor (for AAV2, 3) and integrin (which has been disputed, for AAV2); hepatocyte growth factor receptor (also known as c-Met, for AAV2 and 3, AAV receptor (for AAV1, 2, 5, 6, 8 and 9), and platelet-derived growth factor which is also modified by sialic acid (for AAV5) [32, 34]. However, the major receptors of AAV7 and 8 have not yet been identified, not to mention the comparative expression of these receptors in knee joint tissues [32, 36]. Future studies identifying the receptors for AAV7 and AAV8 and examining the expression level of these primary and secondary receptors in arthritic joints may provide a more holistic understanding of the causes of better transduction efficiency via AAV2, 7, and 8 in cartilage.

At present, with the utilization of gene therapy, there are many preclinical activities for vector development for future OA trials. For instance, the world's first two clinical trials using retrovirus as a carrier to treat OA have been approved by the Korean authorities. To avoid potential carcinogenic activities after intra-articular injection, the human chondrocytes transduced with *TGF-β1*-expressing retrovirus are irradiated before mixing with normal allogeneic human chondrocytes. Phase 1 of both clinical trials revealed that there are no serious adverse effects by this method [37, 38]. However, due to the low copy number integration of the retroviral vector, the expression of *TGF-β1* is limited. Compared with other viral vectors, AAV vector can enter the deeper layers and transduce chondrocytes in the corresponding positions, indicating that AAV may also be a promising strategy of cartilage repair and regeneration in OA. This property is particularly important in treatment of OA, because ECM metabolism and density changes of chondrocytes are considered to be of vital importance for the pathophysiology of OA. Recent studies have found that intra-articular gene-delivery of interleukin-1 receptor antagonists using AAV vectors provides meaningful benefits in the equine forelimb OA model [39, 40]. In addition, phase I trials of gene therapy using AAV2.5, listed on Clinicaltrials.gov (Identifier: NCT02727764), have also been initiated to test the safety of AAV-mediated over-expression of interleukin-1 receptor antagonist on human OA phenotype [7, 41]. Moreover, in this study, we found that in addition to AAV2, AAV7, and AAV8 can also penetrate deeper joint cartilage and transduce chondrocytes *in vivo*, which provides more candidate serotypes for future basic research and clinical trials of OA.

At present, the research on AAV viruses mainly focus on its application and optimization for gene transfer and the following host immune response [32, 42], but the influence of AAV capsid protein on biological functions of different tissues and organs is still unclear, one of the reasons for the uncertain future of AAV vector in clinical applications. Here we found that AAV6 has a significant impact on cartilage integrity, AAV5 and 7 promote the degradation of Col2; and AAV9 may increase the expression of ADAMTS-4, but reduce the production of MMP-19. These results suggest that the application of different serotypes may cause discrepancies in the biological functions of arthritic chondrocytes, therefore more attention should be paid to these changes when using corresponding AAV serotypes for gene transfer to joint tissues.

After being activated by a variety of inflammatory stimuli, inflammasomes can further induce pyroptosis, leading to the programmed death of corresponding cells. It is generally known that NLRP3 inflammasomes contribute to excessive increases in various cytokines with inflammatory diseases. *In vitro* study of synovial tissues from OA patients has revealed that NLRP3 inflammasomes are implicated in the inflammation and pyroptosis, whereas inhibition of NLRP3 results in a large reduction of cytokines caused by pyroptosis, such as Caspase-1. Thus, pyroptosis may be pivotal for the OA pathogenesis, therefore representing a promising therapeutic target [43]. In this study, we found that AAV2, 5, 7 and 8 had no significant effect on the pyroptosis of chondrocytes, indicating intra-articular injection of AAV-mediated gene delivery to chondrocytes did not trigger chondrocyte pyroptosis.

There were, however, several limitations to this study. Firstly, AAV-mediated gene transfer has been proven to maintain long-term expression in many tissues, including arthritic joints of rats and mice [10]. In the present study, we only observed and analyzed AAV-mediated gene delivery of eGFP in arthritic cartilage 30 days after intra-articular injection; but the mechanisms underlying this phenomenon have not been explored yet, which may require further study. Moreover, eGFP expression in synovium and menisci and its corresponding effects have not been investigated. In addition, transduction of AAV usually activates the host’s immune response [32]. It is yet unclear whether the intra-articular injection of AAV has any impact on the activation of the innate and adaptive immune response in mice.

## Conclusions

In conclusion, our findings demonstrate that eGFP protein can be successfully expressed in mouse knee chondrocytes by intra-articular injection of all tested AAV vectors. AAV2 had better transduction efficiency and comprehensive score, and therefore may be the potential ideal candidate AAV vector for local gene transfer to the cartilage of knee joint, enabling chondrocytes located in the deep layer of articular cartilage to be infected smoothly without unintended damage.

## Supporting information

S1 File (DOCX)
