# Supplementary material for: Comparative intra-articular gene transfer of seven adeno-associated virus serotypes reveals that AAV2 mediates the most efficient transduction to mouse arthritic chondrocytes
Source: PLoS One. 2020 Dec 15;15(12):e0243359. doi: 10.1371/journal.pone.0243359 (PMC7737971; doi:10.1371/journal.pone.0243359)

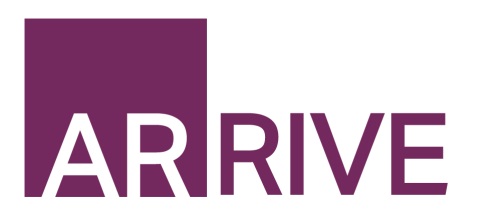


The ARRIVE Guidelines Checklist

Animal Research: Reporting In Vivo Experiments

Carol Kilkenny^1^, William J Browne^2^, Innes C Cuthill^3^, Michael Emerson^4^ and Douglas G Altman^5^

*^1^The National Centre for the Replacement, Refinement and Reduction of Animals in Research, London, UK, ^2^School of Veterinary Science, University of Bristol, Bristol, UK, ^3^School of Biological Sciences, University of Bristol, Bristol, UK, ^4^National Heart and Lung Institute, Imperial College London, UK, ^5^Centre for Statistics in Medicine, University of Oxford, Oxford, UK.*

|  | | ITEM | RECOMMENDATION | Section/ Paragraph |
| --- | --- | --- | --- | --- |
| 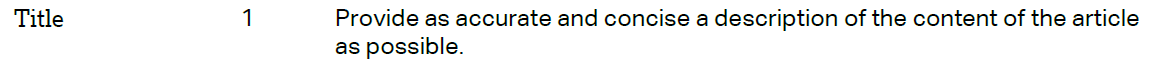 | | | Title |  |
| 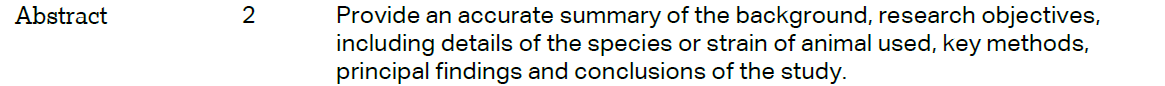 | | | Abstract |  |
| INTRODUCTION | | |  |  |
| 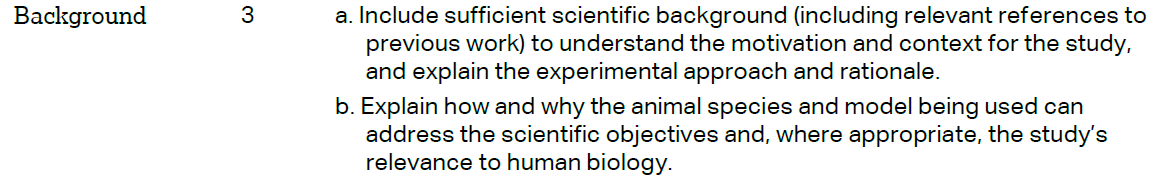 | | | Paragraph 1-2  Paragraph 1-5 |  |
| 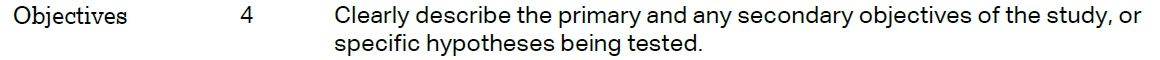 | | | Paragraph 1-2 |  |
| METHODS | | |  |  |
| 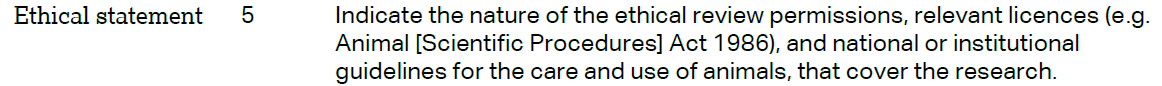 | | | Paragraph 2 |  |
| 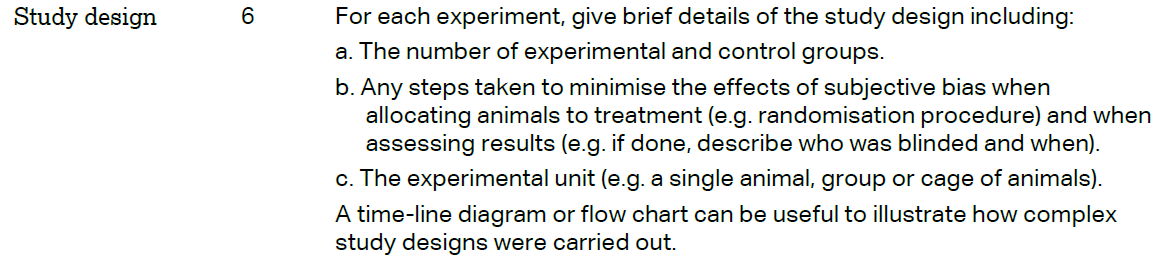 | | | Paragraph 3  Paragraph 2-7  Paragraph 3  Figure 1 |  |
| 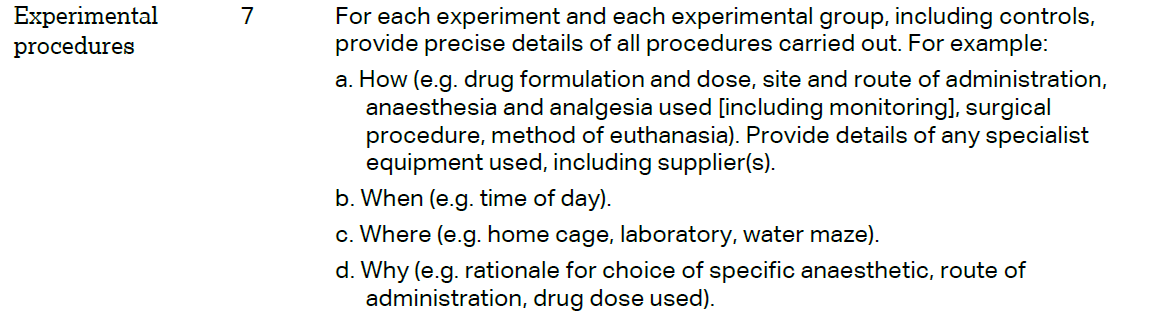 | | | Paragraph 2-8 |  |
| 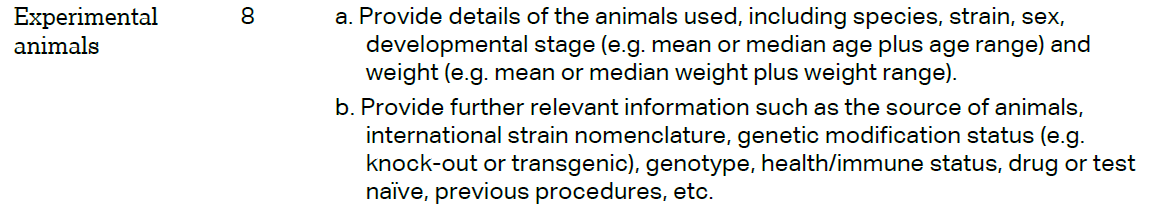 | | | Paragraph 2 |  |

The ARRIVE guidelines. Originally published in *PLoS Biology*, June 2010^1^

| 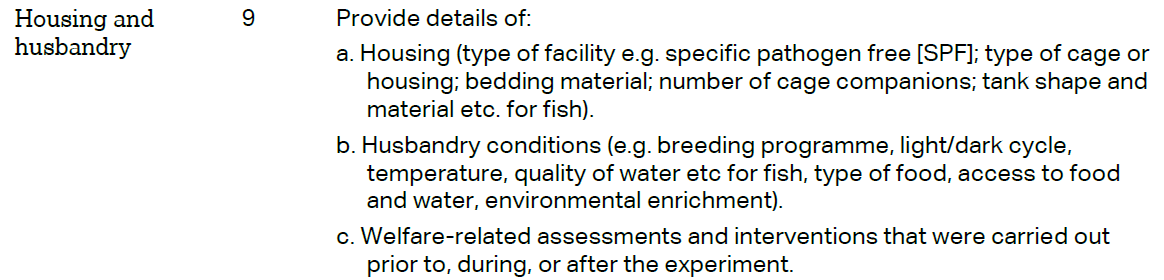 | Paragraph 2 | |
| --- | --- | --- |
| 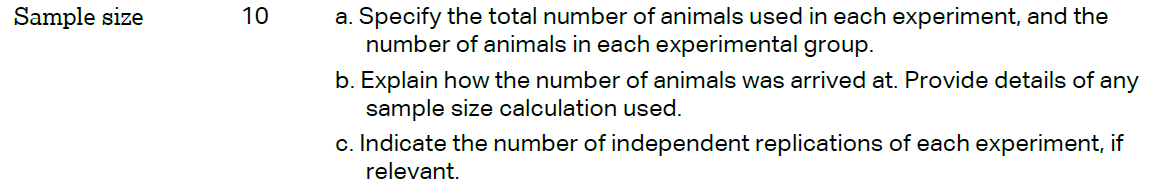 | Paragraph 3 | |
| 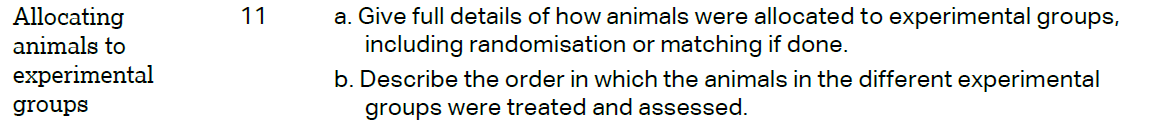 | Paragraph 3-4 | |
| 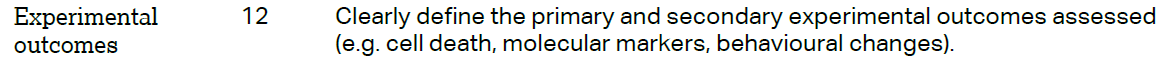 | Paragraph 5-7 | |
| 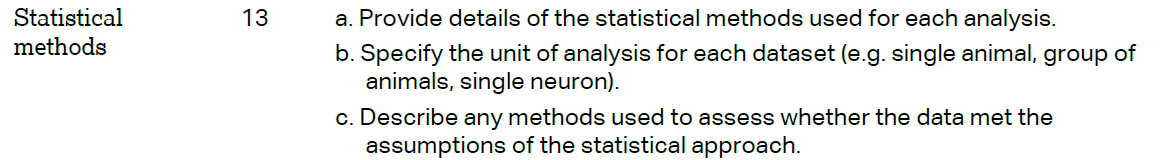 | Paragraph 8 | |
| RESULTS |  | |
| 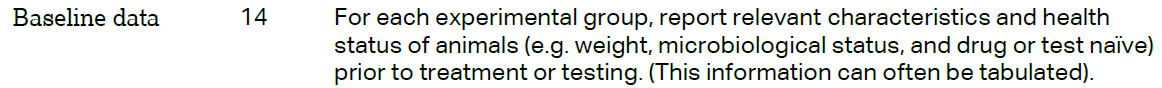 | Methods  paragraph 2 | |
| 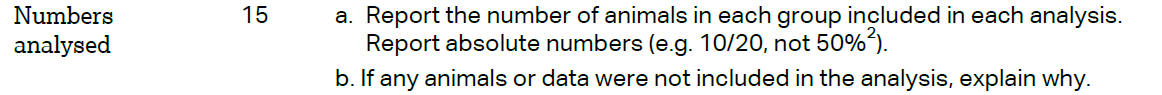 | Methods  paragraph 3-4 | |
| 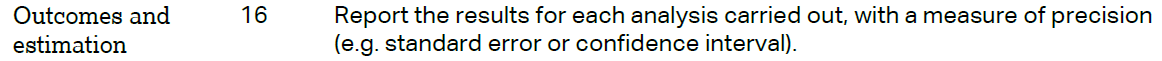 | Paragraph 1-7 | |
| 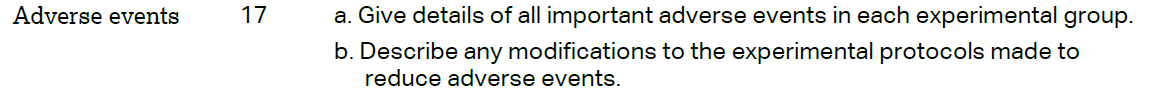 | Paragraph 2 | |
| DISCUSSION |  | |
| 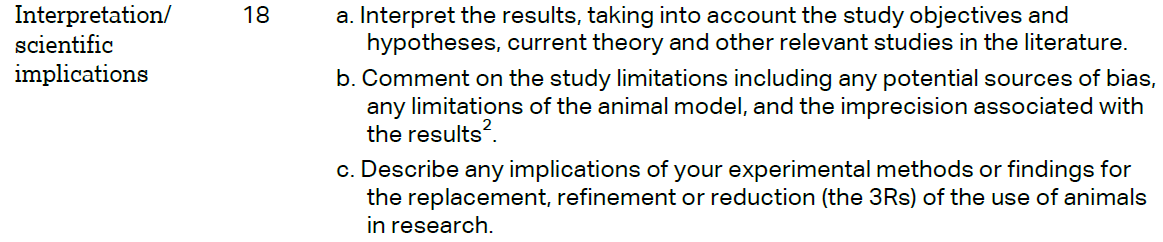 | Paragraph 1 & 7 | |
| 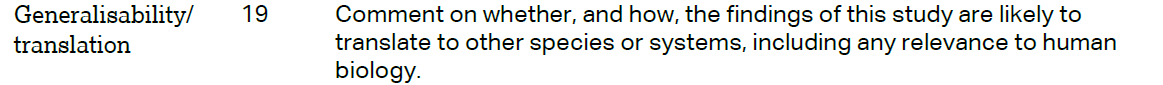 | Paragraph 1 & 2 | |
| 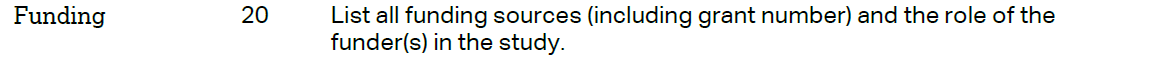 | | Author Contributions  Paragraph 4 |


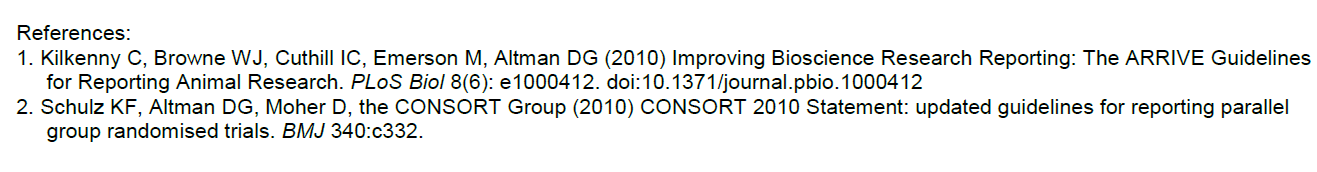

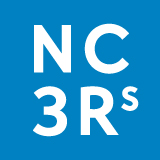

Supplement: S1 File — (DOCX) [file pone.0243359.s001.docx]
